# Antimicrobial usage assessment and the factors associated among small-scale household dairy farms in a district of southern India

**DOI:** 10.5455/javar.2025.l911

**Published:** 2025-05-07

**Authors:** Shwetha Prabhu, Rinila Das, Arun Kharate, Ajith M. Nayak, Navya Vyas

**Affiliations:** 1 Department of Global Public Health Policy and Governance, Prasanna School of Public Health, Manipal Academy of Higher Education, Manipal, Karnataka, India; 2 Department of Veterinary Public Health and Epidemiology, Veterinary College, Nandinagar, Bidar, India; 3 Department of Renal Replacement Therapy and Dialysis Technology, Manipal College of Health Professions, Manipal Academy of Higher Education, Manipal, Karnataka, India

**Keywords:** Antimicrobial resistance, veterinary public health, dairy farms, food security, disease control

## Abstract

**Objective:**

The study was primarily conducted to assess antimicrobial usage, associated factors, and animal health management practices in small-scale household dairy farms in a district of southern India.

**Materials and Methods:**

A total of 330 dairy farmers participated in the study, and single-stage cluster sampling was performed, followed by probability proportional to size sampling. A semi-structured, validated questionnaire and a checklist were used to collect the data.

**Results:**

Only a few dairy farmers knew about antimicrobials (33%) and antimicrobial resistance (AMR) (10.9%). All 330 dairy farmers were using antimicrobials. The factors that influenced dairy farmers’ decisions to use antimicrobials for their cattle were veterinarian advice (100%), para-veterinarian advice (96.3%), peer influence (31.2%), and previous experience of using antimicrobials (12.4%). None of them were aware of the drug withdrawal period and followed it. Significant differences in completing the full course of antimicrobial treatment as prescribed have been observed with increasing levels of education (*p* < 0.001).

**Conclusion:**

Despite the wide usage of antimicrobials in dairy farms, there was a significant lack of knowledge among dairy farmers regarding antimicrobials and AMR. The study emphasizes the need for targeted educational interventions to improve farmers’ understanding of antimicrobial use and resistance, promote responsible practices, and enhance animal health management.

## Introduction

Antimicrobial resistance (AMR) is a global health concern [1] that threatens the effectiveness of antibiotics, posing a substantial risk to both human and animal health. Misuse and overuse of antimicrobials are the main drivers in developing drug-resistant pathogens [2]. The low cost and easy accessibility of antimicrobial medications are among the factors contributing to their irrational usage [2].

On dairy farms, antimicrobials are primarily used to treat respiratory, reproductive, and mastitis infections [3]. Antibiotics are the most often prescribed and used antimicrobials worldwide [4]. Globally, 63,151 ± 1,560 tons of antibiotics are administered yearly to livestock [5]. Antibiotics were administered by unauthorized personnel, such as para-veterinarians, unauthorized practitioners, and farmers [6]. The improper use of antibiotics in food animals was identified as one of the main contributing factors to the rise in AMR by the United Nations (UN) General Assembly [7]. It is estimated that 0.7 million deaths worldwide are caused by antibiotic resistance (ABR) every year. That number is predicted to increase to 10 million by 2050 [8], resulting in a US$100 trillion economic loss [9].

AMR’s extensive impact on Sustainable Development Goal 3 (SDG 3—Good health and well-being) is profound. As AMR escalates, the cost of treatments rises, making universal health coverage increasingly unattainable for many countries [10]. For SDG 1 (No Poverty), AMR could push an additional 28.3 million people into extreme poverty by 2050 due to high treatment costs and chronic infections [10]. For SDG 2 (Zero Hunger), AMR’s harm to animals’ livelihoods and broader food security. Regarding SDG 8 (Decent Work and Economic Growth), the rise in mortality and morbidity from AMR will reduce the labor supply, potentially decreasing global economic output by 1%–3% by 2030, resulting in losses of up to USD 3.4 trillion [10].

Organizations such as the World Health Organization (WHO), Food and Agriculture Organization, and World Organization for Animal Health have advocated for methods to decrease the usage of antimicrobials in food animals, particularly those medications recognized as critically important for human health [1]. The WHO suggests reducing the quantity of medically necessary antimicrobials used in food animals and completely prohibiting AMU in animals to promote growth and prevent illness [1].

The animal health and farming sectors consume a substantial amount of antibiotics, contributing significantly to the emergence and spread of AMR worldwide [11]. In Southeast Asia, the production of food animals is shifting to more intensive and integrated systems to meet the growing demand worldwide. These systems often rely more heavily on antibiotics to maximize animal productivity and health, leading to the emergence and spread of AMR [12]. In food animals, the neglect of drug withdrawal periods leads to products with antibiotic residues [13].

In India, a nation with a booming dairy industry, the challenge of AMR is particularly pronounced, given its status as one of the world’s largest consumers of antibiotics in animal agriculture [14]. In 2017, India was among the top 10 nations consuming the highest number of veterinary antibiotics [15]. By 2030, India is projected to rank among the top five nations in the world in terms of the quantity of antibiotics consumed [16]. Antimicrobial Stewardship programs are being adopted at the national, state, and local levels in several countries to reduce the rising prevalence of AMR [17]. The availability of antibiotics over the counter in India without a prescription and the direct sale of medications to farmers present significant gaps in antibiotic stewardship [18,19].

In India, it is estimated that 70 million households depend on dairy cattle for their livelihood [20]. Good animal management practices in the livestock sector must be improved [21]. Practices, including the non-therapeutic or unreasonable use of antibiotics in lactating cows, are uncontrolled and unregulated on small-scale dairy farms, which lack infrastructure and quality control [19,20,22]. Irrespective of the size of the dairy farm, dairy farmers lack knowledge about antibiotics and the rationale behind their use [19]. In India, efforts have been made to reduce the spread of ABR nationally. The Food Safety and Standards Authority of India has revised the tolerance limits for antibiotics in foods originating from animals [23]. Current work is implemented to address critical gaps in knowledge and practice by conducting a comprehensive assessment of antimicrobial usage practices among small-scale house-hold dairy farms. A thorough exploration of this issue will help safeguard the health of both animals and humans while ensuring the sustainability of India’s dairy industry.

## Materials and Methods

### Ethical approval

Ethical clearance was obtained from the Kasturba Medical College and Kasturba Hospital Institutional Ethics Committee (Reference No IEC2: 642/2023). Permission was also obtained from the state’s milk producers’ union. Written informed consent was obtained from the participants, and the confidentiality of the study participants was maintained throughout the study.

### Study design and target population

A cross-sectional study was conducted in a southern Indian district from January to June 2024. The target population was dairy farmers who provided milk to the collection societies.

### Data collection tool

A semi-structured, validated questionnaire and observational checklist were used to collect the data from the dairy farmers. The questionnaire and an observational checklist were developed and validated by subject experts. The questionnaire was developed in both English and the local language of the district where the study was carried out. The main purpose of the questionnaire and checklist was to assess antimicrobial usage, associated factors, and animal health management practices in dairy farms.

### Sampling technique

Single-stage cluster sampling followed by probability proportional to size sampling was used to select the participants for the study. A list of milk collection societies was obtained from the milk producers’ union. Each milk collection society was considered a cluster, and a list of dairy farmers was obtained from these societies. Then, probability proportional to size sampling was performed. A total of 330 small-scale household dairy farmers participated in the study.

### Statistical analysis

Data were entered in Microsoft Office Excel 2019. The data were exported and analyzed using Jamovi 2.4.14.0. Sociodemographic characteristics, knowledge, antimicrobial usage, and animal health management practices were analyzed using descriptive statistics. The *Chi*-square and Fisher’s exact tests assessed the association between demographic characteristics and knowledge and practice related to antimicrobial usage.

## Results

### Sociodemographic information

A total of 330 dairy farmers participated in the study. The mean age of the participants was 51.2, with a standard deviation of 5.64; around 75.8% were females, and 24.2% were males. Most dairy farmers had only completed primary and secondary school education, i.e., 46.7% and 41.8%, respectively. Most participants were full-time farmers (73%) and had been involved in dairy farming for between 1 and 10 years (55.1%) (Table 1). Most, i.e., 55.1%, of the dairy farmers had been involved in dairy farming for 1 to 10 years.

### Knowledge of antimicrobials

Only 33% of dairy farmers knew what antimicrobials were. The primary source of information for dairy farmers on antimicrobials was veterinarians (92.7%), followed by para-veterinarians (62.4%), family/friends (21.1%), and pharmacists (14.6%). Most were unaware of the side effects of antimicrobials (86.4%), and around 10% thought that the antimicrobials used in humans could also be used in cattle. The highest number of dairy farmers were unaware of the potential risks associated with excessive antimicrobial usage in dairy farming (97%). None of them were aware of the drug withdrawal period (Table 2).

**Table 1.** Sociodemographic information of the participants (*n =* 330).

Variables	Categories	Frequency *n* (%)
Age group (in years)	Mean: 51.2	
	SD: 5.64	
	21–30	1 (0.3%)
	31–40	9 (2.7%)
	41–50	154 (46.7%)
	51–60	152 (46.1%)
	61–70	14 (4.2%)
Gender
	Male	80 (24.2%)
	Female	250 (75.8%)
Education level
	Illiterate	2 (0.6%)
	Primary School	154 (46.7%)
	Secondary School	138 (41.8%)
	Intermediate	33 (10%)
	Undergraduate	1 (0.3%)
	Diploma	2 (0.6%)
Type of family
	Joint family	166 (50.3%)
	Nuclear family	164 (49.7%)
Occupation
	Full-time farmer	241 (73%)
	Part-time farmer	89 (27%)

### Knowledge of AMR

Only dairy farmers knew about AMR (10.9%) and that AMR could develop in animals (6.4%). The significant sources of information on AMR were family/friends (58.3%), followed by veterinarians (36.1%), para-veterinarians (11.1%), and pharmacists (8.3%). Most of them did not know that inappropriate and frequent antimicrobial usage causes the emergence of resistant pathogens (97.9%). Most were unaware that antimicrobial usage in cattle could affect humans and that AMR could be transferred from humans to animals and vice versa (99.7%) (Table 3).

### Antimicrobial usage and associated factors

All 330 dairy farmers were using antimicrobials. All of them used antibiotics for treatment (100%) and anthelmintics for treatment (74.9%), prevention (3.3%), and growth promotion (21.8%) among cattle. The primary sources of obtaining antimicrobials for their cattle were veterinarians (99.3%), followed by para-veterinarians (96.3%) and over-the-counter purchases (20.30%). The majority of dairy farmers practiced checking the expiry date before giving stored antimicrobials (83.3%) and had practiced completing the entire course of antimicrobials as prescribed (93.9%). Nearly all sought advice from veterinarians (99.1%) and para-veterinarians (98.2%) to ensure proper dosage when administering antimicrobials to their cattle. Most dairy farmers did not have the practice of increasing the dose of antimicrobials and frequency of administration if cattle did not show any sign of recovery (99%). None of them were following the drug withdrawal period.

**Table 2.** Knowledge about antimicrobials (*n =* 330).

Variable	Category	Frequency *n* (%)
Knowledge of what antimicrobials are	Yes	109 (33%)
	No	221 (67%)
Knowledge of the side effects of antimicrobials	Yes	45 (13.6%)
	No	285 (86.4%)
Antimicrobials used in humans can be used in cattle as well
	Yes	33 (10%)
	No	296 (89.7%)
	Somewhat	1 (0.3%)
Knowledge about potential risks associated with excessive antimicrobial usage in dairy farming
	Yes	4 (1.2%)
	No	320 (97%)
	Somewhat	6 (1.8%)
Knowledge about the drug withdrawal period	No	330 (100%)
Knowledge about any guidelines regarding proper antimicrobial usage in cattle farming, or have received any training	No	330 (100%)

**Table 3.** Knowledge about AMR (*n =* 330).

Variables	Category	Frequency *n* (%)
Knowledge about AMR
	Yes	36 (10.9%)
	No	294 (89.1%)
Knowledge about inappropriate and frequent antimicrobial use causes the emergence of resistant pathogens
	Yes	7 (2.1%)
	No	323 (97.9%)
Knowledge of AMR developing in animals
	Yes	21 (6.4%)
	No	309 (93.6%)
Knowledge about the use of antimicrobials in cattle affects humans
	Yes	1 (0.3%)
	No	329 (99.7%)
Knowledge about AMR can be transferred from humans to animals and vice versa
	Yes	1 (0.3%)
	No	329 (99.7%)
Knowledge about sharing the same environment with cattle is a risk for AMR
	Yes	2 (0.6%)
	No	328 (99.4%)

**Figure 1.**
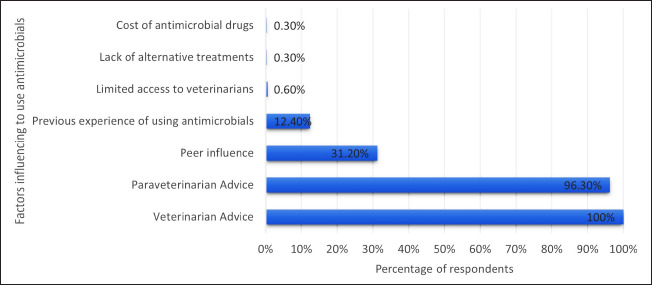
Factors influencing dairy farmers’ decision to use antimicrobials for cattle.

The factors that influenced dairy farmers’ decisions to use antimicrobials for their cattle were veterinarian advice (100%), para-veterinarian advice (96.3%), peer influence (31.2%), and previous experience of using antimicrobials (12.4%). Additional factors influencing the decision to use antimicrobials included limited access to veterinarians (0.6%), the lack of alternative treatments (0.3%), and the cost of antimicrobials (0.3%) (Fig. 1).

**Figure 2.**
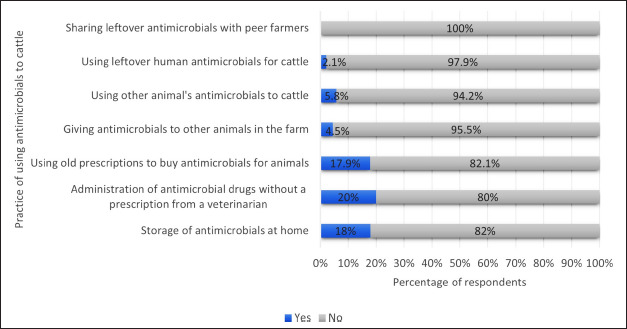
Dairy farmers’ practice of using antimicrobials for their cattle (*n =* 300).

Most dairy farmers were not storing antimicrobials at home (82%). Around 80% of them did not administer the antimicrobials without a prescription from the veterinarian. About 17.9% of them were using old prescriptions to buy animal antimicrobials. Reasons for using old prescriptions were peer influence (81.4%), travel expenses (18.6%), and limited veterinarian access (13.6%). About 2.1% of them were using human antimicrobials for cattle. None of the dairy farmers were sharing leftover antimicrobials with peer farmers (Fig. 2).

Dairy farmers who were completing the full course of antimicrobial treatment as prescribed were among the age group less than 50 (95.2%), among females (93.6%), among those who had completed primary school education (95.4%), among those who had been involved in dairy farming for less than 12 years (95.1%), among those who were part-time farmers (96.6%), and among those who had dairy animals as their source of extra income (95.3%). Dairy farmers who were completing the full course of antimicrobial treatment as prescribed had knowledge of antimicrobials (94.5%), those who think antimicrobials have no side effects (97.8%), those who think antimicrobials used in humans can be used in cattle as well (93.9%), those who stop giving antimicrobials if cattle feel better (80.5%), those who administer antimicrobial drugs without a prescription from a veterinarian or para-veterinarian (90.9%), and those who were using old prescriptions to buy antimicrobials for animals (89.8%) (Table 4). As the level of education increased, significant differences in completing the full course of antimicrobial treatment as prescribed was observed (<0.001).

### Animal health management practices

Dairy farmers reported that mastitis (97.2%) and reproductive problems (76.6%) were the common health problems among their cattle. Only a few reported fevers (3.3%) and diarrhea (0.3%) as common health problems. All 330 dairy farmers vaccinated their cattle against foot-and-mouth disease (100%), lumpy skin disease (100%), and brucellosis (76.9%). Although all were vaccinating their cattle, 76% of them did not give booster vaccinations to their cattle. None of the dairy farmers were maintaining cattle health status and vaccination records. Only a few dairy farmers stated that cattle faced adverse reactions related to vaccination (1.2%) and faced challenges regarding access to veterinary services (3.3%). Most dairy farmers do not actively seek updates on the latest developments and recommendations regarding cattle vaccines and vaccination strategies (99.7%). None of the dairy farmers quarantined newly acquired cattle or had a specific procedure to ensure cattle were up to date for vaccination before introducing them to the existing herd.

**Table 4.** Association between sociodemographic characteristics, knowledge of antimicrobials, and practice related to the completion of a full course of antimicrobial treatment as prescribed (*n =* 330).

Variable	Category	Complete the full course of antimicrobial treatment as prescribed		*p*-value
		Yes *n* (%)	No *n* (%)	
Age (in years)
	<50	120 (95.2%)	6 (4.8%)	0.437^a^
	≥50	190 (93.1%)	14 (6.9%)	
Gender
	Male	76 (95.0%)	4 (5.0%)	0.792^b^
	Female	234 (93.6%)	16 (6.4%)	
Education
	Illiterate	0	2 (100%)	0.004^b^
	Primary school	146 (95.4%)	7 (4.6%)	
	≥ Secondary school	164 (93.7%)	11 (6.3%)	
Experience (number of years involved in dairy farming)
	<12	174 (95.1%)	9 (4.9%)	0.332^a^
	≥12	136 (92.5%)	11 (7.5%)	
Occupation type
	Full-time farmer	224 (92.9%)	17 (7.1%)	0.300^b^
	Part-time farmer	86 (96.6%)	3 (3.4 %)	
Reason for keeping dairy animals
	Primary income	109 (91.6%)	10 (4.4%)	0.180^a^
	Extra income	201 (95.3%)	10 (4.7%)	
Knowledge of what antimicrobials are
	Yes	103 (94.5%)	6 (5.5%)	0.766^a^
	No	207 (93.7%)	14 (6.3%)	
Antimicrobials have side effects
	Yes	44 (97.8%)	1 (2.2%)	0.332^b^
	No	266 (93.3%)	19 (6.7%)	
Antimicrobials used in humans can be used in cattle as well
	Yes	31 (93.9%)	2 (6.1%)	1.000^b^
	No	279 (93.9%)	18 (6.1%)	
Aware of the potential risks associated with excessive antimicrobial usage in dairy farming
	Yes	4 (100%)	0	1.000^b^
	No	6 (100.0%)	0	
	Somewhat	300 (93.8%)	20 (6.2%)	
Administering antimicrobial drugs without a prescription from a veterinarian or para-veterinarian
	Yes	60 (90.9%)	6 (9.1%)	0.249^a^
	No	250 (94.7%)	14 (5.3%)	
Using old prescriptions to buy antimicrobials for animals
	Yes	53 (89.8%)	6 (10.2%)	0.144^a^
	No	257 (94.8%)	14 (5.2%)	
Ensuring a full course of antimicrobial treatment by seeking advice from para-veterinarians
	Yes	6 (100%)	0	1.000^b^
	No	304 (93.8%)	20 (6.2%)	

Note: ^a^ Association computed using *Chi*-square test; b association computed using Fisher’s exact test.

**Figure 3.**
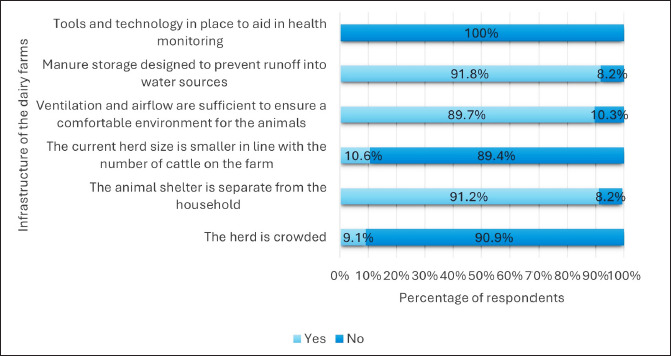
Infrastructure of the dairy farms (*n =* 300).

**Figure 4.**
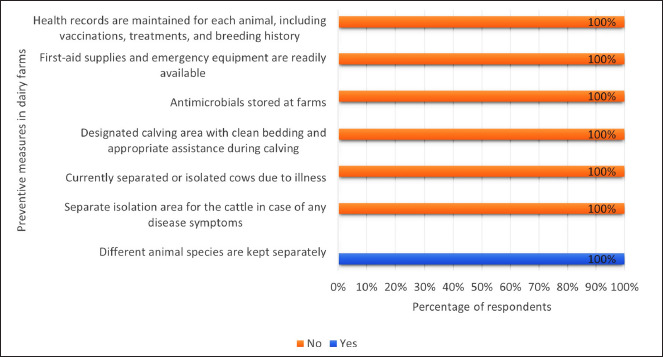
Preventive measures in dairy farms (*n =* 300).

Most dairy farmers had animal shelters separate from the household (91.2%). Only 9.1% of them had the herd crowded. About 89.7% had sufficient ventilation and airflow to ensure a comfortable environment for the animals. Around 91.8% had manure storage designed to prevent runoff into water sources. None of them had tools and technology to aid in health monitoring (Fig. 3). All 330 dairy farmers had kept different animal species separate. None had cows currently isolated due to illness, nor did they store antimicrobials on their farms. Additionally, they lacked first-aid supplies and emergency equipment and maintained no health records for each animal (Fig. 4).

## Discussion

The prudent use of antimicrobials is essential for maintaining their effectiveness in treating infectious diseases. A cross-sectional study was conducted to assess antimicrobial usage and to identify the factors influencing it on small-scale household dairy farms. A total of 330 dairy farmers participated in the study. In the current study, only 33% of dairy farmers were aware of what antimicrobials are, and significant sources of information on the subject were veterinarians (92.7%) and para-veterinarians (62.4%). The findings of a study conducted in rural Peru reported that only 22.4% of farmers knew what antibiotics are [24]. None (100%) of the dairy farmers knew of the drug withdrawal period and were not following that. The study findings were concordant with a study conducted in Assam and Haryana, where only 2% knew the term withdrawal period [25]. The findings of a study conducted in Telangana indicated that only 20% of respondents were aware of the withdrawal periods. Among them, only 10% followed the withdrawal periods [26]. Previous studies conducted in Jhunjhunu District, Rajasthan, and rural Peru revealed that only 33.7% and 33.1% were aware of the drug withdrawal period, respectively [17,24]. A study in Nigeria indicated that only 18.7% of Fulani pastoralists observe a withdrawal period among dairy cows [27].

The current study indicated that only 10.9% of dairy farmers had knowledge of AMR, and their major sources of information on the subject were family/friends (58.3%) and veterinarians (36.1%). Only a few, i.e., 6.4%, knew that AMR can develop in animals. The findings were discordant with those of a study conducted in Rajasthan, where 55.4% of the farmers were aware of AMR, and the source of awareness of AMR was doctors/veterinarians (34.1%) [21].

All, i.e., 100% of dairy farmers, were using antimicrobials for their cattle. Most of the dairy farmers obtained antimicrobial drugs from veterinarians (99.3%), para-veterinarians (96.3%), and a few from over-the-counter purchases (20.3%). The findings were discordant with the findings of a study conducted in eastern Haryana, which revealed that the source of veterinary consultancy among small farmers was 50% of them consulted veterinarians, 30.36% of them consulted para-veterinarians, 12.50% of them procured antibiotics through over-the-counter sales, and 7.14% of them obtained it through milk vendors on the occurrence of disease regularly [28]. Another study conducted in Telangana revealed that for cattle farmers, the most reliable and favored source for obtaining antibiotics from veterinary pharmacies based on para-veterinarians’ followed by veterinarian prescriptions and over the counter medication [26]. In the study conducted in eastern Turkey, 48% of the cattle farmers did not consult veterinarians to administer antibiotics [29]. The current study revealed that all dairy farmers used antibiotics for treatment (100%). The study findings were concordant with a study that reported that small dairy farmers utilized antibiotics therapeutically (98.21%), with only marginal subtherapeutic use (1.79%) found [28].

The study findings indicate that the factors influencing dairy farmers’ decision to use antimicrobials for their cattle were veterinarian advice (100%), para-veterinarian advice (96.3%), peer influence (31.2%), previous experience of using antimicrobials (12.4%), and limited access to veterinarians (0.6%). A previous study had similar findings that reported veterinarian advice (100%) and previous experience (100%) as the major factors for using antimicrobials [30]. Another study conducted in eastern Turkey revealed that 64% of the farmers took advice from other farmers about antibiotic use [29].

Most dairy farmers did not have the practice of increasing the dose of antimicrobials and frequency of administration if cattle did not show any sign of recovery (99%). In a study conducted in eastern Turkey, findings were different; 45% of the farmers increased the dosage when animals did not show signs of recovery [29]. A significant proportion (83.3%) of the dairy farmers checked expiry dates before giving antimicrobials to cattle. The findings were different in a study where 70% of the cattle farmers were not checking the expiry date of antibiotics [26].

The study findings indicated that mastitis (97.2%) and reproductive problems (76.6%) were the major health problems among cattle. The findings of this study were in line with the study conducted in smallholder dairy farms in four regions of India, which reported that in all four regions, mastitis and reproductive problems were the most common diseases among dairy animals [22]. Although all dairy farmers vaccinated their cattle against foot-and-mouth disease (100%), lumpy skin disease (100%), and brucellosis (76.9%), booster vaccinations were not administered in 76% of the cases. This study’s findings were concordant with another study conducted in Jhunjhunu District, Rajasthan, India, which indicated that 77.8% of the farmers vaccinated their animals [21]. The limitation of the study was its reliance on self-reported information from dairy farmers, which could have been affected by recall bias.

## Conclusion

Despite the wide usage of antimicrobials in dairy farms, there was a significant lack of knowledge among dairy farmers regarding antimicrobials and AMR. Mastitis and reproductive problems were commonly reported health problems. Farmers often rely on veterinarians and para-veterinarians to obtain antimicrobials. However, there was lack of awareness and non-compliance with drug withdrawal periods. The study emphasizes the need for targeted educational interventions to improve farmers’ understanding of antimicrobial use and resistance, promote responsible practices, and enhance animal health management. There is a need to include training sessions for farmers, educational efforts by veterinarians, and comprehensive studies involving other livestock sectors.

## Acknowledgments

The authors would like to thank the Manipal Academy of Higher Education, the State’s Milk Producers’ Union, and the Department of Animal Husbandry and Veterinary Services for providing the administrative support for conducting this study. The authors would also like to acknowledge the participation and cooperation of dairy farmers in the study.

## List of abbreviations

ABR, antibiotic resistance

AMR, antimicrobial resistance

AMS, antimicrobial stewardship

FSSAI, Food Safety and Standards Authority of India

FAO, Food and Agriculture Organization

WOAH, World Organization for Animal Health

UN, United Nations

SDG, sustainable development goal

WHO, World Health Organization.
